# Mental health services in Ukraine during the early phases of the 2022 Russian invasion

**DOI:** 10.1192/bjp.2022.170

**Published:** 2023-02

**Authors:** Ryunosuke Goto, Irina Pinchuk, Oleksiy Kolodezhny, Nataliia Pimenova, Norbert Skokauskas

**Affiliations:** Department of Pediatrics, The University of Tokyo Hospital, Japan; Institute of Psychiatry, Taras Shevchenko National University of Kyiv, Ukraine; Regional Centre for Children and Youth Mental Health and Child Welfare – Central Norway, IPH, Norwegian University of Science and Technology, Norway; and Chair, Child and Adolescent Psychiatry Section, World Psychiatric Association

**Keywords:** Epidemiology, low and middle income countries, military psychiatry, in-patient treatment, human rights

## Abstract

**Background:**

In February 2022, Russia began its invasion of Ukraine. War increases the demand for mental healthcare among affected populations, but with devastating losses across the nation, it is unclear if Ukrainian mental health services are able to meet the needs of the people.

**Aims:**

We aimed to evaluate the state of Ukrainian in-patient mental health services, which remains the backbone of the nation's psychiatric services, early in the 2022 Russian invasion.

**Method:**

We conducted a nationwide cross-sectional study on Ukrainian in-patient mental health facilities during the 2022 Russian invasion. Using an online questionnaire, we obtained responses from the heads of 32 in-patient mental health facilities across Ukraine, representing 52.5% of all in-patient mental health facilities in the nation. We gathered information on hospital admissions, staff, humanitarian aid received and the additional needs of each facility.

**Results:**

Hospital admissions were reduced by 23.5% during the war (April 2022) compared with before the war (January 2022). Across facilities, 9.6% of hospital admissions in April 2022 were related to war trauma, with facilities reporting percentages as high as 30.0%. Facilities reported reductions in staff, with 9.1% of total medical workers displaced and 0.5% injured across facilities. One facility reported that 45.6% of their total medical workers were injured. Although facilities across Ukraine have received humanitarian aid (such as medical supplies, food, volunteers), they reported additionally needing equipment as well as more staff.

**Conclusions:**

The mental health service structure in Ukraine has been severely damaged during the 2022 invasion, with staff shortages despite a significant number of hospital admissions related to war trauma.

## Background

During humanitarian crises, the demand for mental health services among people living in affected regions increases drastically.^1,2^ This ranges from children, who are at risk of delayed socioemotional development with exposure to armed conflict^3^ and need socioemotional support, to civilian and non-civilian populations at risk of numerous psychiatric problems stemming from psychological distress because of trauma exposure.^4^ Furthermore, war disrupts care for pre-existing health problems, for both displaced people and people staying in affected regions.^5,6^

## Mental health services in Ukraine prior to the 2022 invasion

Even prior to the recent invasion, Ukraine was a country with significant mental healthcare needs and limited resources.^7^ For instance, whereas 12.4% of adults in Ukraine had symptoms consistent with a diagnosis of depression, only 3.2% of individuals with depression received treatment.^8^ Furthermore, Ukraine continues to rely heavily on in-patient services to provide mental healthcare. Unlike many low- and lower-middle-income countries that tend to put more emphasis on community-based mental health services, Ukraine, along with several other nearby countries from the former Soviet Bloc, relies heavily on in-patient psychiatric services (this is a relic of the so-called ‘Semashko model’ of healthcare). There have been recent efforts to shift Ukraine's mental healthcare to a more community-centred approach, although they have been stymied by the COVID-19 pandemic.^9^

To address its needs for mental healthcare, Ukraine adopted a mental health action plan on 10 October 2021.^9^ However, the war, which began on 24 February 2022, made these plans virtually impossible to achieve: according to Ukraine's Ministry of Health, as of 21 May, the Russian forces have damaged 627 healthcare facilities, of which 105 have been completely destroyed.^10^ In addition, at least 12 physicians have been reported to be killed and 47 seriously wounded.^11^ These numbers may only be the tip of the iceberg. Without doubt, the war has crippled many aspects of Ukraine's healthcare system, despite the significant mental healthcare needs of the people. Failing to provide continued care to meet these needs could exacerbate the evolving public health crisis. Available evidence suggests that the current mental health support structure may not be able to fully address the new and pre-existing mental health needs of the Ukrainian people amidst the war. For instance, in a recent study, we found that Ukrainian helpline staff demonstrated signs of compromised mental health amidst the 2022 Russian invasion of Ukraine, with 68% of the 25 interviewed staff burned out and 40% screening positive for depression.^12^

## Aims

The mental health of Ukrainian residents is at stake. Despite multiple calls for action to address Ukraine's mental health needs^2,13^ little is known about the viability of in-patient mental health services in Ukraine, which remains the backbone of the nation's psychiatric services, amidst the 2022 Russian invasion. Thus, using data on mental health facilities across the nation we aimed to evaluate the viability and needs of mental health facilities in Ukraine as of May to June 2022.

## Method

To assess the state of mental health services in Ukraine as of May to June 2022, we conducted a cross-sectional study by distributing an online questionnaire to the heads of in-patient mental health facilities in Ukraine. Ukraine has 25 regions, each of which has one leading psychiatric hospital. In addition, many regions have several smaller psychiatric hospitals other than the main hospital. We contacted the heads of each of the main psychiatric hospitals in all 25 regions of Ukraine via online messaging. Some of the heads of the facilities were recruited via phone, after which they were sent an online message. In addition to completing questionnaires about their own hospitals, the chiefs of the main hospitals were asked to distribute the questionnaires to the smaller psychiatric hospitals in the region. Of 61 psychiatric hospitals in Ukraine, we were able to recruit 37 in our study.

Data were collected from 2 May to 2 June 2022, and the head of each facility provided informed consent to participate in the study. Although the study participants were not directly involved in the questionnaire design, they were involved in the data collection and recruitment of additional participants.

The study was approved by the ethics committee of Taras Shevchenko National University of Kyiv's Institute of Psychiatry (No. 5/19.04.2022). All procedures contributing to this work comply with the ethical standards of the relevant national and institutional committees on human experimentation and with the Helsinki Declaration of 1975, as revised in 2008.

Heads of facilities provided information on the mental health services at their facilities before the start of the 2022 invasion (January 2022) and during the invasion (April 2022). They were asked about the number of in-patient beds, total hospital admissions, hospital admissions related to war trauma (referring to any mental illnesses secondary to exposure to war), number of staff (psychiatrists, nurses, junior nurses, psychologists and social workers), number of injured workers, number of displaced workers, whether their facility was directly occupied by Russian forces, the humanitarian aid they received (medical supplies, food, etc.) and the additional needs that they may have.

Comparisons of data before and during the war were conducted using Wilcoxon signed-rank tests. To compute percent changes in hospital admissions and percentages of hospital admissions related to war trauma across facilities we weighted these proportions by the number of hospital admissions per facility in January 2022. Similarly, percentages of injured workers and displaced workers were weighted by the number of total medical workers per facility in January 2022.

To visualise the data, we created proportional symbol maps of percentages of displaced workers (out of total medical workers), per cent reductions in number of hospital admissions and percentages of hospital admissions related to war trauma. In addition, we mapped the humanitarian aid received during the 2022 Russian invasion and the additional needs of the facilities as of May to June 2022. For anonymity, the data used for these maps were aggregated by region. These maps were overlaid on a map of territorial occupation by Russia, based on manually curated military event reports indicating whether the administrative centre or other major city of each district are/were occupied by the Russian forces by 2 May 2022.^14^ In our analyses on hospital admissions (number of hospital admissions before and after the start of the invasion and per cent of hospital admissions related to war trauma), we excluded facilities that did not have any hospital admissions prior to the start of the invasion. All analyses were conducted using R version 4.1.1.

## Results

In this cross-sectional study, we obtained responses from heads of 32 facilities (25 leading regional facilities and 7 smaller regional facilities; response rate, 86.5%), which represent 52.5% of all psychiatric hospitals in Ukraine. The average number of in-patient beds per facility was 394.6 beds, and there were fewer hospital admissions during the war (April 2022) compared with before the war (January 2022) (324.8 *v*. 424.4 per month, Wilcoxon signed-rank test *P* = 0.002), with a 23.5% reduction in hospital admissions across facilities (Table 1).

**Table 1:** Basic characteristics^a^

Facility characteristics	Value (*n* = 32)
Number of in-patient beds, mean (SD)	394.6 (306.1)
Hospital admissions in January 2022, mean (SD)	424.4 (270.9)
Hospital admissions in April 2022, mean (SD)	324.8 (282.7)
Reduction in hospital admissions, %	23.5
Hospital admissions related to war trauma, %	9.6
Number of psychiatrists, mean (SD)	
In January 2022	35.8 (29.0)
In April 2022	31.0 (24.4)
Number of nurses, mean (SD)	
In January 2022	143.5 (120.2)
In April 2022	125.1 (115.2)
Number of junior nurses, mean (SD)	
In January 2022	161.1 (134.7)
In April 2022	138.5 (110.5)
Number of psychologists, mean (SD)	
In January 2022	12.1 (34.9)
In April 2022	5.5 (5.2)
Number of social workers, mean (SD)	
In January 2022	1.8 (2.1)
In April 2022	1.5 (1.7)
Injured workers out of total medical workers, %	0.5
Displaced workers out of total medical workers, %	9.1
Facilities evacuated because of Russian occupation, *n*	2
Facilities that received humanitarian aid, *n*	
Medicines	27
Other medical supplies	22
Food	21
Other humanitarian aid received	–^b^
Facilities in need of the following, *n*	
Medicines	25
Other medical supplies	18
Food	16
Other needs	–^c^

a. Percentages are weighted percentages.

b. Humanitarian aid also received by facilities: volunteers, bedding, mattresses, underwear, blankets, towels, hygiene items (data not shown).

c. Facilities also in need of the following: staff, information on evacuated patients, generator, mattresses, beds, linen, detergents (data not shown).

**Fig. 1:**
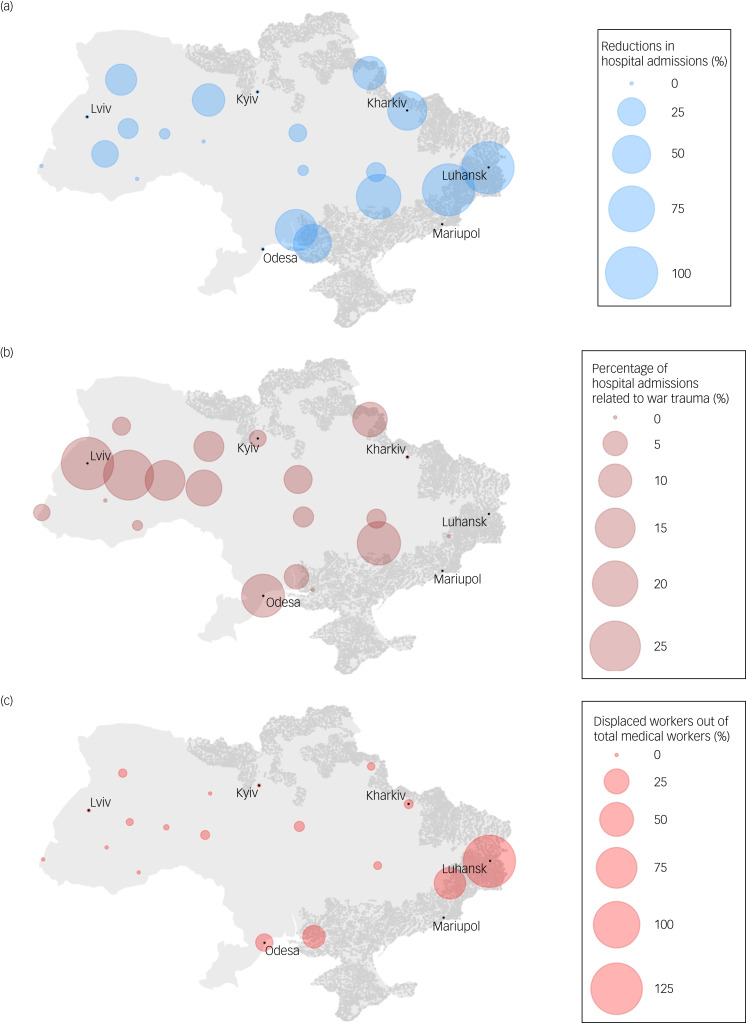
Proportional symbol maps of changes in mental health services in Ukraine during the 2022 Russian invasion. Proportional symbol maps were created for (a) percent of hospitalisations related to war trauma (%), (b) reductions in number of hospitalisations (%) and (c) displaced workers out of total medical workers (%). Each circle represents the percentages aggregated by region. Shaded areas (in grey) represent regions that have been or are under Russian occupation as of 2 May 2022. All hospitals with more hospitalisations in April than January 2022 are shown to have no reductions in hospitalisations (0%). Note that percentages of displaced workers out of total medical workers could exceed 100% as some workers may not have been medical workers. Statistics for regions with unavailable data are not shown.

Although facilities that experienced reductions in hospital admissions were dispersed across Ukraine, facilities that had the most reductions were concentrated in the Eastern regions, which were occupied by the Russian forces (Fig. 1(a)).

Across facilities, 9.6% of hospital admissions in April 2022 were related to war trauma (Table 1). Facilities with the largest percentages of hospital admissions related to war trauma were located in regions not under direct Russian occupation, with facilities reporting percentages as high as 30.0% (Fig. 1(b)).

There were fewer nurses (125.1 *v*. 143.5 per facility, Wilcoxon signed-rank test *P* = 0.01), junior nurses (138.5 *v*. 161.1 per facility, *P* = 0.03) and psychologists (5.5 *v*. 12.1 per facility, *P* = 0.03) during the war compared with before the war. We did not find strong evidence of fewer psychiatrists (31.0 *v*. 35.8 per facility, *P* = 0.24) and social workers (1.5 *v*. 1.8 per facility, *P* = 0.09) during the war compared with before the war.

Across facilities, 9.1% of the total medical workers were displaced and 0.5% were injured, and one facility reported that 45.6% of the health workers were injured. Facilities with displaced workers were largely concentrated around the eastern regions, some of which were directly occupied by the Russian forces (Fig. 1(c)). Patients and staff of two hospitals were evacuated as Russia occupied the territory these hospitals were located in.

**Fig. 2:**
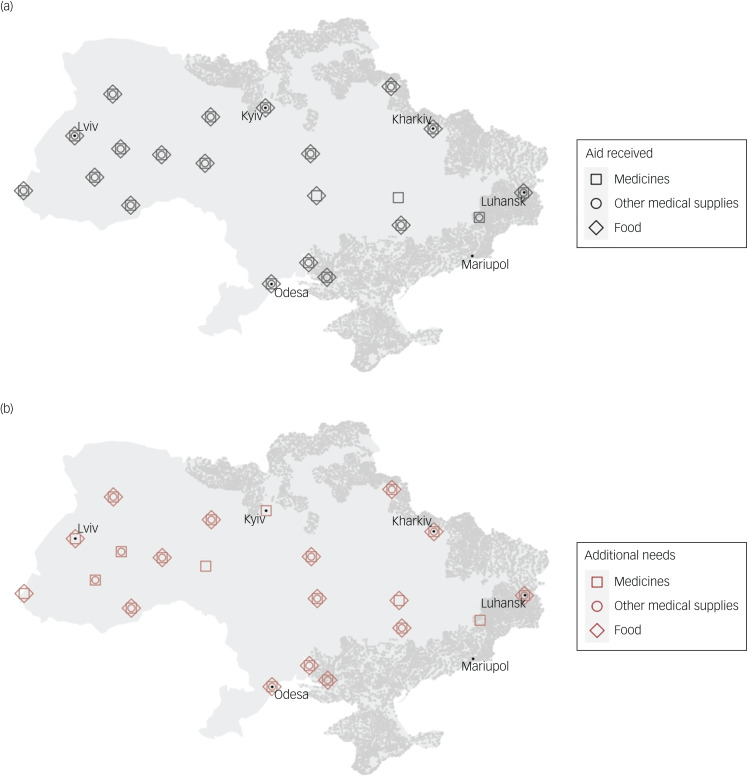
Maps of the needs and humanitarian aid received during the 2022 Russian invasion. Maps were created for (a) humanitarian aid received by mental health facilities and (b) additional needs of mental health facilities, aggregated by region. Shaded areas (in grey) represent regions that have been or are under Russian occupation as of 2 May 2022. Other aid received and needs are shown in Table 1. Statistics for regions with unavailable data are not shown.

Although many facilities received humanitarian aid, such as medical supplies, food, volunteers and linen (Fig. 2(a)), facilities reported additional needs for this equipment (Fig. 2(b)) as well as more staff (Table 1). Facilities that received aid or needed additional equipment were dispersed across the nation (Figure 2). Summary statistics of other facility-level data are available in Table 1.

## Discussion

### Main findings

During the 2022 Russian invasion, many Ukrainian mental health facilities experienced decreases in hospital admissions and staff shortages. Although facilities that experienced the largest decreases in hospital admissions were located in regions directly occupied by Russia, the facilities outside these regions also saw decreases in hospital admissions. This could be partially the result of the decreased demand because of the displacement of the residents, but it could also represent a compromised mental health service structure in Ukraine because of the war. We found that the needs of mental health facilities in Ukraine were largely unmet, with many facilities requiring additional medical supplies, food and linen, despite the humanitarian aid that had been provided to facilities throughout the nation. Importantly, facilities also stressed the need for increased staffing, likely stemming from the fact that many workers had been displaced or injured. Meeting these needs is critical amidst the increased mental health burden of the conflict-affected population,^15^ as represented by our finding that a large portion of hospital admissions in facilities across the nation were related to war trauma.

### Interpretation of our results

Even before the 2022 invasion, psychiatric disorders such as depression, anxiety and post-traumatic stress disorder (PTSD) were prevalent among Ukrainian residents.^8^ For instance, a study on more than 2000 internally displaced adults in Ukraine found that in 2016, following the start of the Russo-Ukrainian War in 2014, the prevalence of depression, anxiety and PTSD was 22%, 17% and 32%, respectively.^16^ With the war, the Ukrainian population is at an even higher risk of mental health disorders. This is supported not only by our finding that a significant portion of hospital admissions across Ukraine was related to war trauma but also by previous research on conflict-affected populations. For instance, in a meta-analysis of mental disorders in conflict settings, the prevalence of mental disorders (depression, anxiety, PTSD, bipolar disorder and schizophrenia) was found to be greater than 20% among conflict-affected residents.^15^ Taken together with our findings, mental health services in Ukraine need to be scaled to meet the increased mental health needs of the conflict-affected Ukrainian population. With the needs of mental health facilities largely unmet, more aid will be necessary to overcome this public mental health emergency.

### Recommendations

One solution may be to seek the help of general healthcare practitioners and non-mental healthcare specialists. The World Health Organization and United Nations High Commissioner for Refugees have designed the mental health Gap Action Program Humanitarian Intervention Guide (mhGAP-HIG) specifically for this purpose,^17^ and primary healthcare clinics and general hospitals could use the mhGAP-HIG to meet the needs for mental healthcare among affected populations. The feasibility of the mhGAP for medical and non-medical specialists has been supported in multiple low- and middle-income countries including Ukraine,^7,18^ and thus the mhGAP-HIG may also show promise in conflict-affected Ukraine (which could, if continued post-conflict, help expand Ukraine's community-based mental healthcare). However, based on our finding that the mental health workforce has been severely damaged across the nation, the non-mental health workforce may also have suffered, especially in regions directly under attack by the Russian forces.

In addition, remote-access psychiatric services have been proposed as alternatives to face-to-face aid at mental health facilities.^2^ However, based on our previous finding that Ukraine-based helpline staff have experienced symptoms of burnout and compromised mental health early in the war (for example out of 25 helpline staff, 17 had burnout and 10 screened positive for depression),^12^ even this may not be a sufficient alternative, at least domestically. Remote mental health support from outside Ukraine should be considered, as telemedicine and remote consulting are increasingly recognised as an effective tool for specialists to provide healthcare services without the logistical challenges of being based in an area affected by conflict.^19^

### Limitations

Our findings should be interpreted in light of several limitations. First, there may have been measurement bias, as our data was collected through a self-reported questionnaire by heads of psychiatric hospitals. Second, although our data collection protocol was designed to be as representative as possible of the in-patient mental health services across Ukraine, the fact that some facilities did not participate in our study may limit the representativeness of our sample. However, data collection during an active conflict is extremely challenging, and to our knowledge, our study is the first to provide such comprehensive information on the mental health service structure in Ukraine during the 2022 Russian invasion. Finally, the data on hospital admissions related to war trauma were collected from heads of mental health facilities but future studies should thoroughly evaluate the needs of the Ukrainian people amidst the invasion through data directly collected from civilian and non-civilian populations.

### Implications

Ultimately, our study highlights the significant damage Ukraine's mental health service structure suffered amidst the 2022 Russian invasion. Facilities in regions that are or have been under Russian occupation saw significant decreases in hospital admissions and staffing, and two facilities were under direct Russian occupation. Facilities outside regions under Russian occupation had many hospital admissions related to war trauma, which may reflect the mental health needs of internally displaced Ukrainians. Although humanitarian aid has been provided to facilities across the nation, needs are largely unmet, with many facilities stressing the need for more staff. Further studies are needed to assess which interventions are effective in addressing the increased need for mental health support amidst the invasion.

## Data Availability

Data used for this study are not publicly available.
